# Predictors of Pregnant Women's Decision to Opt for Cesarean Section in Romania

**DOI:** 10.7759/cureus.69185

**Published:** 2024-09-11

**Authors:** Mihaela Corina Radu, Loredana S Manolescu, Sebastian M Armean, Irina Prasacu, Joeri Vermeulen, Melania Elena Pop Tudose, Cosmin Medar, Razvan D Chivu

**Affiliations:** 1 Nursing, Carol Davila University of Medicine and Pharmacy, Bucharest, ROU; 2 Obstetrics, Emergency County Hospital, Ploiești, ROU; 3 Virology, Microbiology, and Parasitology, Carol Davila University of Medicine and Pharmacy, Bucharest, ROU; 4 Pharmacology, Toxicology, and Clinical Pharmacology, Iuliu Hațieganu University of Medicine and Pharmacy, Cluj-Napoca, ROU; 5 Fundamental Sciences, Carol Davila University of Medicine and Pharmacy, Bucharest, ROU; 6 Healthcare, Erasmus Brussels University of Applied Sciences and Arts, Brussels, BEL; 7 Public Health, Biostatistics, and Medical Informatics, Vrije Universiteit Brussel, Brussels, BEL; 8 Obstetrics and Gynecology, Carol Davila University of Medicine and Pharmacy, Bucharest, ROU; 9 Clinical Laboratory of Radiology and Medical Imaging, Clinical Hospital "Prof. Dr. Theodor Burghele", Bucharest, ROU; 10 Public Health Sciences, Carol Davila University of Medicine and Pharmacy, Bucharest, ROU

**Keywords:** childbirth education, midwives, torch, pregnant women, cesarean

## Abstract

Introduction: In Romania, the latest official report indicates that more than half of the births (80,890 cases, representing 52.88% of the total) are performed by cesarean, a rate significantly higher than the World Health Organization (WHO) recommendation of 15-20%. This study aims to identify the predictors associated with women's decisions to opt for cesarean in Romania.

Materials and methods: An analytical cross-sectional observational study was conducted in the general population of Romania. The study was carried out over the course of 2023, with a total duration of four months. During this period, researchers targeted pregnant women from various regions of the country, regardless of their place of residence, age, or education level. The primary data collection tool was a self-administered online questionnaire, distributed via Google Forms, an accessible and efficient platform that allows for automatic response collection. The questionnaire was distributed online, particularly on social media platforms frequented by pregnant women, such as Facebook, Instagram, and TikTok.

Results: A total of 1,301 participants were validated. Socio-demographic and clinical factors significantly influence women's decisions to give birth by cesarean. Among these participants, 435 expressed a preference for cesarean delivery. Key predictors include fear of pain and concern for the child's health. Fear of pain at birth is the first predictor in Romanian women to choose cesarean (OR=2.09; 95% CI: 1.62-2.68). Concerns about the child's health do not increase the likelihood of opting for a cesarean.

Conclusion: By utilizing valuable resources such as midwives and implementing strategies like birth plans, significant contributions can be made toward reducing the cesarean rate and improving the childbirth experience for women worldwide.

## Introduction

Globally, the rate of cesarean has been on the rise in recent decades. According to the World Health Organization (WHO), the percentage of cesareans continues to increase, currently accounting for more than one in five (21%) of all births [1]. The WHO recommends an optimal cesarean rate of 15-20%. However, this number is projected to keep increasing in the next decade, with nearly one-third (29%) of all births expected to be performed through cesarean by 2030 [1]. Recent studies reveal that Romania has the highest cesarean rate in Europe at 46.9% [1].

In 2020, over 1.12 million cesareans were performed in the European Union member states, according to Eurostat data. In Romania, cesarean on maternal request is one of the indications set out in the national clinical guide for cesarean, which is adopted as secondary legislation by the Ministry of Health. The guide defines cesarean on request as an intervention performed based on obstetric and medical indications, but also at the explicit request of the mother, before the beginning of labor. In contrast, a cesarean performed during labor is termed an emergency cesarean. The guide grants obstetricians-gynecologists the authority to perform cesareans on request "after appropriate and documented advice" [2].

In Romania, there are no data regarding the number of cesareans performed on maternal request. The current health information system does not collect these data, and the coding and classification systems used do not include a code for this category of interventions.

According to the National Institute of Statistics (NSI), in 2020, Romania recorded 201,849 births [3]. The cesarean rate has gradually increased in recent years, along with the cesarean-to-vaginal birth ratio in Romanian hospitals. In 2020, approximately 152,961 births (including 80,890 cesareans) were reported to the National School of Public Health (NSPH), by 196 hospitals/obstetrics and gynecology departments. These births were covered by compulsory health insurance (174 public healthcare units and 22 private units). Overall, more than half of the births (80,890 cases, representing 52.88% of the total) were cesareans, while 72,071 cases, representing 47.1%, were reported as vaginal births.

NSPH collects a minimal set of data for each admission from public and private hospitals funded by compulsory health insurance as part of the health services reimbursement system based on the Diagnosis Related Groups (DRG) classification system. This system is similar to the International Classification of Diseases (ICD), where diagnoses are classified into classes and subclasses. However, unlike the ICD, the DRG system employs an additional criterion for classification, namely, the cost of resources consumed for patient care. Through the DRG system, patients can be simultaneously classified based on both pathology and the cost of care, providing the ability to associate patient types with hospital expenses incurred.

Therefore, births/cesareans in private hospitals paid for by private health insurance or directly by patients are not included, which may explain the discrepancy of 48,888 births between the number of births reported by NSI (201,849 births in 2020) and the number of births reported by hospitals to NSPH (152,961 births in 2020).

The increase in the number of births by cesarean has not been accompanied by significant benefits to maternal or perinatal health. According to WHO, a cesarean is a surgical procedure that can prevent maternal and neonatal mortality when used in medically indicated situations. The ascending trend of cesarean has not been associated with significant maternal or perinatal outcomes. Evidence suggests that beyond a certain threshold, an increase in cesarean rates may be linked with increased maternal and perinatal morbidity. Cesarean is accompanied by short- and long-term risks potentially affecting the health of the woman, the child, and future pregnancies. Moreover, high cesarean rates are associated with substantial healthcare costs [4].

With the rapid increase in cesarean rates, an increasing number of women face the dilemma of how to give birth. There are conflicting reports regarding the safety of cesarean [5]. Despite advances in medicine and anesthesia, there remains a significant risk; cesarean has a 3.01 times increased risk of maternal mortality compared to vaginal birth [6-9].

A significant concern and costly consequence of cesarean delivery is the limited number of subsequent cesarean sections that a woman can safely undergo. Repeated cesarean interventions can lead to severe complications such as uterine ruptures, uterine atony, placenta previa, placenta accreta, as well as an increase in surgical adhesions [10]. The rising number of cesarean sections cannot be attributed solely to medical indications but also to non-medical factors, such as the individual preferences of women or healthcare providers, as well as the lack of adequate prenatal education for women and their families [11].

The purpose of this article is to investigate the factors that may influence the decision to opt for a cesarean, considering that there is no available data in Romania regarding the cause of increased cesarean rates.

## Materials and methods

Study design, selection, and participants

A cross-sectional observational analytical survey was conducted in Romania within the general population. For rigorous design, we followed the Strengthening the Reporting of Observational Studies in Epidemiology (STROBE) guidelines in our observational study. The STROBE checklist is included in Appendix A.

Pregnant women from the general population of Romania, residing in various counties, including the capital, constituted the target group. The inclusion criteria for participants required them to be at least 18 years old and to hold Romanian citizenship. The participants' financial status was assessed based on their occupational status, classifying them as having a stable job or being unemployed. The level of education was categorized according to the highest level of education attained: primary school, high school/post-secondary, and university/postgraduate studies.

Survey questionnaire and data collection

The self-administered questionnaire was used as the primary data collection tool in this study due to its efficiency and accessibility. It was designed using Google Forms, a cloud-based survey software that allows for the easy administration and automatic collection of responses. To ensure national coverage and reach as many participants as possible, the questionnaire was distributed as a link in various social media groups frequented by pregnant women across the country. The platforms chosen for distribution included popular social networks such as Facebook, Instagram, and TikTok, which are widely used in Romania and provide considerable exposure.

The distribution campaign was conducted over a period of four months, from April to July 2023, to allow sufficient time for collecting a representative number of responses and to include women at different stages of pregnancy. Participants were informed in advance about the purpose of the survey, including the research objectives and how the collected data would be used. This step was essential to ensure transparency and to adhere to ethical principles in research.

Completion of the questionnaire was considered an act of informed consent on the part of the respondents, indicating that they understood and accepted the terms and conditions of participation in the study. To ensure the quality and integrity of the data collected, all questions in the questionnaire were mandatory, and the questionnaire could only be submitted once all questions were answered. This approach allowed for the collection of a complete and coherent dataset, essential for the subsequent analysis of the results.

The questionnaire was structured into four parts. The first part, the general data section, included five questions with simple answers, collecting general demographic information. The second part, the obstetric profile section, comprised five questions with straightforward responses, focusing on collecting obstetric history and related information. The third part, the knowledge and information section, consisted of eight questions aimed at understanding how pregnant women were informed about various aspects of pregnancy, childbirth, and postpartum care. In the final part of the questionnaire, pregnant women were asked to agree, disagree, or remain neutral regarding a series of 18 statements related to scientific evidence about vaginal birth and cesarean sections.

The described questionnaire contains statements that address perceptions and attitudes related to vaginal birth and cesarean section, including medical, emotional, and cultural aspects. Each of the questions aims to capture respondents' opinions on the advantages and disadvantages of each type of birth, the impact on the mother and child, as well as the factors influencing decisions regarding the method of delivery.

After the initial design, the questionnaire was tested on a small sample to identify any ambiguities, formulation issues, or comprehension problems. Reliability was measured using Cronbach's alpha coefficient (0.8).

The full questionnaire is available in Appendix B.

Statistical analysis

The data collected through the questionnaire underwent a rigorous analysis using two main statistical tools: Microsoft Office Excel and IBM SPSS Statistics for Windows, Version 23.0 (Released 2015; IBM Corp., Armonk, New York, United States). Excel was initially used for organizing and filtering the raw database. For this purpose, the COUNTIFS function was essential, allowing researchers to efficiently filter responses and sort data according to various criteria relevant to the study. This initial data processing stage was crucial to ensure the integrity and quality of the data before applying more complex statistical analyses.

Regarding the variables analyzed, these included socio-demographic characteristics such as age, education level, and occupational status, as well as details concerning the obstetric profile of the participants, including medical history and birth mode preferences. These categorical variables were expressed using descriptive statistics such as means, medians, frequencies, and percentages to provide a detailed and clear picture of the study population.

One of the primary objectives of the analysis was to investigate the choices of pregnant women regarding the mode of delivery, whether vaginal or cesarean. This was defined as the main dependent variable, and the analysis aimed to explore how this choice was influenced by various socio-demographic and obstetric variables. Each dependent variable was compared with the general group variables to identify possible patterns or trends.

To establish the relationships between variables and determine the statistical significance of these relationships, chi-squared tests were employed. These tests were used to analyze the association between categorical variables and allowed for the identification of significant correlations between the different characteristics of pregnant women and their preferences regarding the mode of delivery.

In the bivariate analysis, the variables associated with the mode of birth included age, place of origin, level of education, marital status, employment, number and type of previous births, gestational age, number of prenatal visits, participation in prenatal education courses, information about the mode of birth, the importance of early contact with the newborn, the "golden hour," breastfeeding, and information about midwifery services and the need for access to a midwife during pregnancy, birth, and postpartum. The "golden hour" represents the period immediately after birth when the newborn and the mother establish the first contacts and affective bonds, a period considered crucial for the development of the emotional bond and attachment between mother and child.

For further analysis, the primary dependent variable was entered into a logistic regression to identify predictors of pregnant women's decisions regarding the mode of delivery. The independent factors selected as predictors were fear of pain at birth influencing the mode of delivery, perception that cesarean babies are healthier than those born vaginally, and performance of the tests recommended, including the TORCH panel (tested for TORCH (*Toxoplasma gondii*, human immunodeficiency virus (HIV), syphilis, hepatitis B virus (HBV), hepatitis C virus (HCV), *Listeria monocytogenes*, rubella, cytomegalovirus, herpes simplex virus (HSV)).

The variables were coded with 0 and 1, with the value 1 expressing the occurrence of a certain event, so the goal was to estimate the probability of that event occurring as a function of the values of the independent variables. For all tests, a statistical significance threshold of 0.05 was considered.

Ethical approval

The questionnaire was validated by colleagues and approved by the Ethics Committee of the Emergency County Hospital, Ploiești, Romania (approval number: 41482/09.08.2022). All study procedures adhered to the ethical standards of the Helsinki Declaration. Informed consent was mandatory.

## Results

In this study, a total of 1319 responses were collected. During the data verification process, 18 participants were excluded from the study because they did not meet the minimum age criterion of 18 years. The first part was dedicated to collecting general data, essential for characterizing the group of participants. Thus, the final analysis was conducted on a sample of 1301 respondents, representing a significant number and providing a solid basis for the study's conclusions. Among these participants, 435 expressed a preference for cesarean delivery. This significant percentage of 33% indicates an important trend in the choice of delivery method, reflecting both safety concerns and the social and cultural influences that may shape these decisions.

The majority of women in both groups have higher education. A large proportion of women in both groups live in urban areas and have a stable occupation. Most women are married in both groups. Most women had more than six prenatal consultations, with a higher proportion in the group of women opting for a cesarean. Women without previous births are the majority in the group of women desiring a cesarean. Participation in prenatal education courses is lower in the group of those opting for a cesarean. Women desiring to give birth in a private hospital are more prevalent in the group of women wanting to give birth by cesarean and also consider that they did not receive information from the doctors who monitored their pregnancy, compared to the general group. The socio-demographic characteristics, clinical characteristics, and level of information about pregnancy, childbirth, and breastfeeding received during pregnancy are presented in Table 1.

**Table 1:** Socio-demographic characteristics, clinical characteristics, and level of information about pregnancy, childbirth, and breastfeeding received during pregnancy are presented Welch's t-tests were used if the categorical variable had two categories ANOVA tests followed by post hoc procedures were used if the categorical variable had more than two categories MD: medical doctor

	Category	General group N (%)	Cesarean group n(%)	P-value
		N=1301	n=435	
		(98.71%)	n/N (%)	
Age	18-29 years	689 (52.95)	170 (13.06)	<0.05
	30-39 years	570 (43.81)	248 (19.06)	
	Over 40 years	42 (3.22)	17 (1.3)	
Place of residence	Rural	365 (28.05)	130 (9.99)	<0.05
	Urban	936 (71.94)	305 (23.44)	
Level of education	Primary/middle school	76 (5.84)	16 (1.22)	<0.001
	High school	252 (19.36)	79 (6.07)	
	Higher education	973 (74.78)	340 (26.13)	
Occupational status	Stable occupation	987 (75.86)	362 (27.82)	<0.05
	Occasional/no occupation	314 (24.13)	73 (5.68)	
Marital status	Married	1060 (81.47)	379 (29.13)	<0.05
	Unmarried/divorced	241 (18.52)	56 (4.3)	
Number of prenatal consultations	Under 5	435 (33.43)	82 (6.3)	<0.001
	Over 15	866 (66.55)	353 (27.12)	
Previous births	No previous births	501 (38.5)	240 (18.44)	<0.05
	One or more than one birth	800 (61.49)	195 (14.98)	
Participation in educational programs	Yes	476 (36.58)	115 (8.83)	<0.05
	No	825 (63.41)	320 (24.59)	
Place of childbirth	Public hospital	916 (70.41)	122 (9.37)	<0.05
	Private hospital	340 (26.13)	313 (24.05)	
	Other location	45 (3.46)	0 (0)	
The MD who monitored the pregnancy informed the women about the birth process	Yes, complete information	470 (36.12)	242 (18.85)	<0.05
	Yes, partial information	357 (27.44)	121 (9.3)	
	No discussion with the MD	474 (36.43)	72 (5.53)	
The MD who monitored the pregnancy informed the women about the importance of not separating the mother from the child in the first hours after birth	Yes, complete information	191 (14.68)	70 (5.38)	<0.05
	Yes, partial information	105 (8.07)	42 (3.38)	
	No discussion with the MD	1005 (77.24)	323 (24.82)	
The MD informed the women about breastfeeding and its benefits	Yes, complete information	224 (17.21)	88 (6.8)	<0.05
	Yes, partial information	170 (13.06)	61 (4.68)	
	No discussion with the MD	907 (69.71)	286 (21.98)	

Pregnant women in our study were asked to agree, disagree, or remain neutral regarding a series of statements related to scientific evidence about vaginal birth and cesarean. Logistic regression to identify predictors of pregnant women's decisions to give birth by cesarean revealed that the first predictor in Romanian women to choose cesarean (OR =2.09; 95% CI: 1.62-2.68) was the fear of pain at birth (Table 2).

**Table 2:** Results of logistic regression analysis on women's decision on cesarean *Tested for TORCH (*Toxoplasma gondii*, human immunodeficiency virus (HIV), syphilis, hepatitis B virus (HBV), hepatitis C virus (HCV), *Listeria monocytogenes*, rubella, cytomegalovirus, herpes simplex virus (HSV)) "Yes" coded as 1 indicating the presence of the characteristic or belief "No" coded as 0 indicating the absence of the characteristic or belief B: coefficient; SE: standard error; Wald: Wald test; P: p-value; OR: odds ratio; CI: confidence interval

	B	SE	Wald	P	OR	Lower CI 95%	Upper CI 95%
Fear of birth pain (Yes 1, No 0)	0.737	0.127	33.55	0.000	2.09	1.62	2.68
Cesarean baby is healthier (Yes 1, No 0)	-0.768	0.254	9.16	0.002	0.46	0.28	0.76
Tested for TORCH recommendation (Yes 1, No 0)	-0.274	0.126	4.74	0.029	0.76	0.59	0.97
Constant	-1.02	0.251	0.166	0.684	0.90		

## Discussion

This study examines the factors and predictors associated with cesarean, with a focus on the influence of education and knowledge about childbirth. The findings highlight that the factors associated with women's decision to give birth by cesarean are the socio-demographic and obstetrical profile, as well as the information provided by professionals and the level of knowledge about birth. These factors can limit the available options, influence decisions, and affect women's autonomy.

Among the variables analyzed, fear of birth pain, the perception that babies born by cesarean are healthier, and undergoing TORCH panel tests are all significant factors that influence the choice of cesarean.

As maternal age advances, the likelihood of resorting to surgical intervention for childbirth increases, influenced by a series of physiological and medical factors that emerge with advancing age. Numerous studies have shown that age is considered a risk factor for certain health problems during pregnancy influencing the choice of the birth method [12-16].

The educational level of women is another predictive factor associated with the choice of cesarean delivery as confirmed by other studies [17]. A higher level of education implies a better understanding of the pregnancy and childbirth process, enabling well-educated women to make more informed decisions. Recent research indicates that over 80% of women with higher education chose cesarean [18,19], a phenomenon that may be linked to the fact that a higher level of education is often associated with higher purchasing power and access to private medical services, which are known for higher rates of cesarean.

The results of the study showed that the perception of the risk of prematurity for newborns via cesarean reduces the probability of choosing a cesarean. Therefore, providing frequent and comprehensive information and counseling when discussing a cesarean with pregnant women is essential, as indicated by other studies [20-22]. The perception that a cesarean baby is healthier (p=0.002) does not significantly influence the decision to opt for a cesarean (OR=0.46; 95% CI: 0.28-0.76). In our study, only 8.83% of the respondents who chose cesarean had participated in prenatal courses.

Many literature studies have reached a consensus on the benefits of attending prenatal classes, including a lower risk of cesarean, a reduced frequency of epidural anesthesia, and the adoption of alternative pain management techniques. Women who attended these courses also tend to arrive at the hospital at an advanced stage of labor, adopt exclusive breastfeeding for a longer period, and have a lower risk of developing postpartum emotional distress [23,24].

Providing women with information about the physiology of labor and pain mechanisms is essential for a deep understanding of the birth process. Prenatal care plays a crucial role in educating and informing women, as better knowledge can help reduce the fear associated with childbirth and give them the ability to choose a mode of birth without being influenced by stereotypes related to pain and fear. Through prenatal education, women's knowledge is enhanced, and the number of options available to them increases. The involvement of midwives in the decision-making process is particularly beneficial, as these specialized professionals can facilitate communication and empower women to protect themselves against obstetric violence and unnecessary interventions during pregnancy and childbirth [25-27].

A decisive factor in choosing a cesarean is the fear of pain. The results of another study emphasize the need to provide counseling services for pregnant women who are fearful of childbirth. This usually consists of multiple sessions with an experienced midwife, aimed at having a positive birth experience, regardless of the mode of birth [28].

Women's health education, an essential component of prenatal care, could contribute to reducing the percentage of cesareans in Romania. Midwives play a significant role in counseling and education in healthcare, not only for the woman but also for the family and community. This activity should involve prenatal education and preparation for parenthood and can extend to women's education about sexual and reproductive health and childcare [4].

Numerous studies have shown that women who participate in such childbirth education courses are better prepared to manage anxiety during labor and childbirth. An Australian literature review indicated that antenatal education during pregnancy reduces anxiety during labor and childbirth and increases partner involvement [29]. Research conducted in the United Kingdom found that antenatal courses offered by the British National Health Service led to significant improvements in stress, anxiety, and depression symptoms among pregnant women and their partners [30-32]. A study conducted in Turkey concluded that antenatal education seems to alleviate the fear of childbirth and symptoms of post-traumatic stress disorder after childbirth [33,34]. Meanwhile, a systematic review and meta-analysis of clinical studies conducted in Hungary established that antenatal courses reduce the fear of childbirth, as does the associated use of hypnosis [35]. A study conducted in Poland found that participation in childbirth education courses reduced fear levels during labor and childbirth [36]. A prospective, observational, multicenter study conducted to measure variables in pregnant women who attended antenatal education courses in various health districts in Spain found positive effects on the second stage of labor, reduced rates of episiotomy, and early breastfeeding [37]. A randomized study conducted in Denmark evaluated the effects of an antenatal program in which women in the intervention group reported increased confidence in their ability to manage the birthing process [38]. A Japanese study assessed the positive impact of antenatal courses on the postpartum period and the adaptation of infants at three months after birth [39]. One of the few studies conducted in the Arab world, carried out in Iran, yielded significant results regarding the level of pain experienced by women who participated in the courses [40].

Despite midwifery university education aligning with European standards, in Romania, the insufficient implementation of the midwifery profession and their visibility and accessibility to Romanian women severely restricts women's options. Women typically address the family doctor, who then refers them to an obstetrician, favoring an interventional technical approach. The exclusion of midwives from maternity care and the approach of the pregnancy, birth, and postpartum events as medical events may contribute to elevated cesarean rates, maternal and infant mortality, and births among minors [41,42]. Additionally, the shortage of midwives is a challenge in Romania. This shortage is caused by a series of factors, including the need for a better-defined regulatory framework for the profession, as well as policies and measures that allow midwives to practice at all levels of care: primary, outpatient, and hospital care. Midwives play a crucial role in promoting vaginal births, and their presence in both community and hospital settings can increase trust and improve the quality of prenatal care. The current regulatory framework presents ambiguities in numerous aspects, ranging from malpractice legislation to the methodologies and tools used to evaluate the effectiveness, accessibility, and costs of prenatal care, as well as interventions aimed at improving women's medical knowledge.

The results of this study showed that the decision to give birth by cesarean is related to hospital funding, meaning that women who choose to give birth in the private healthcare system are 50% more likely to opt for a cesarean.

This correlation has been observed in other studies [43-45], suggesting the occurrence of a high number of non-indicated cesareans, primarily in the private sector. In recent years, cesarean has transitioned from being an exclusive method for improving perinatal outcomes to a consumer commodity more common among women with higher purchasing power and higher education [46,47]. Therefore, socioeconomic issues may influence the method of birth, raising concerns about access and equity in healthcare services. Additionally, the choice of a cesarean can be linked to social status, the convenience of scheduling the birth, and the desire to avoid the pain of vaginal delivery. The decision about the mode of birth should be analyzed by multiple healthcare professionals and made in conjunction with the pregnant woman, respecting her autonomy. Proper training for professionals can guide toward understanding cesarean as a practical and safe procedure. In addition, the operation is viewed by several women in labor as a pain reduction mechanism, ignoring perinatal risks [47]. One aspect of decision-making is the request for a cesarean. Requests for maternal cesarean without medical reasons have been reported as a contributing factor to the rising trend of cesarean [48,49]. The concept of "patient choice" is well-accepted among obstetricians [50]. The choice of the method of birth can be influenced by multiple factors, such as cultural values and economic factors, but especially by the information provided during prenatal care [51]. Women's decisions about the mode of birth can be significantly influenced by doctors.

Although a significant percentage of pregnant women have undergone tests for TORCH infections, the results of these tests do not appear to significantly influence the decision on how to give birth (OR=0.76; 95% CI: 0.59-0.97). This phenomenon suggests the possibility that obstetricians may have a predominant role in directing birth-related choices in prenatal care. It is possible that the professionalism and experience of obstetricians could outweigh the impact of TORCH test results in determining the method of delivery. This finding highlights the importance of actively involving both doctors and midwives in pregnancy management, highlighting the need for a holistic and personalized approach to prenatal care for every pregnant woman. The multicenter study "Born in Brazil," which involved 24,940 women, found that 66% preferred vaginal birth at the beginning of pregnancy, but 51.5% ultimately delivered vaginally, indicating a significant decrease in the rate of vaginal births compared to women's initial desires [52]. Although the reasons for this decline in the number of vaginal births are not entirely clear, when women's preferences are compared with the actual number of cesareans, the hypothesis can be formulated that women are not truly autonomous during pregnancy and childbirth, possibly due to the fact that they are not sufficiently informed about the risks and benefits of each type of birth.

The fear of pain is one of the main factors that leads women to opt for a cesarean. Women who do not have access to information about analgesia options for vaginal birth (such as epidurals or other pain management methods) may be more likely to request a cesarean to avoid the intense pain associated with labor. Support from the midwife during labor can reduce anxiety and fear of pain. By offering alternative pain management methods (breathing techniques, movement, emotional support, and, in some cases, administering epidural analgesia), midwives can help reduce the number of cesareans. Developing a birth plan in collaboration with a midwife, offering realistic options to women during labor, would allow the medical team to better understand the preferred choices of women and better meet their needs and desires. Research indicates that women who engage in prenatal education and/or create a birth plan have a 26-98% likelihood of giving birth vaginally [53,54]. Education on infectious diseases transmittable to the fetus is crucial [55,56].

Strengths and limitations

A notable strength of this study is that participants completed the questionnaire in the comfort of their own homes, ensuring confidentiality and anonymity. The efficiency of data collection and analysis allowed researchers to obtain results within a relatively short time frame. The study is relevant because it can serve as a basis for implementing more effective health policies, given the significant increase in the cesarean rate in many countries over the past decades. This increase prompts a reevaluation of the underlying reasons and implications for maternal and infant health. Improving the management of cesarean can bring significant benefits to maternal and infant health, consolidating effective global practices and policies.

However, it is important to note that there were certain limitations. The study sample consisted of 1301 childbearing women, this is a relatively large sample, and the results may not be generalizable to the entire country. The questionnaire responses were collected without direct interaction with the participants, and the environment in which the questionnaire was completed could not be controlled. This introduces the possibility that some responses may be incomplete.

Another potential limitation of the study could be the accessibility of smartphones and the internet. According to a Kepios analysis, there were 18.06 million internet users in Romania at the start of 2024 with the internet penetration at 91.6%.

## Conclusions

Involving women in birth-related decisions, supported by adequate prenatal education and open dialogue with healthcare professionals, is essential for promoting higher standards in maternal care. By utilizing valuable resources such as midwives and implementing strategies like birth plans, significant contributions can be made toward reducing the cesarean rate and improving the childbirth experience for women worldwide.

## Appendices

Appendix A

**Table 3:** STROBE statement: checklist of items that should be included in reports of observational studies *Give information separately for cases and controls in case-control studies and, if applicable, for exposed and unexposed groups in cohort and cross-sectional studies The STROBE checklist is best used in conjunction with the explanation and elaboration article. This article and separate versions of the checklist for cohort, case-control, and cross-sectional studies are available at www.strobe-statement.org STROBE: Strengthening the Reporting of Observational Studies in Epidemiology

	Item no.	Recommendation
Title and abstract		
	1	(a) Indicate the study's design with a commonly used term in the title or the abstract
		(b) Provide in the abstract an informative and balanced summary of what was done and what was found
Introduction		
Background/rationale	2	Explain the scientific background and rationale for the investigation being reported
Objectives	3	State specific objectives, including any prespecified hypotheses
Methods		
Study design	4	Present the key elements of the study design early in the paper
Setting	5	Describe the setting, locations, and relevant dates, including periods of recruitment, exposure, follow-up, and data collection
Participants	6	(a) Cohort study? Give the eligibility criteria and the sources and methods of selection of participants. Describe the methods of follow-up. Case-control study? Give the eligibility criteria and the sources and methods of case ascertainment and control selection. Give the rationale for the choice of cases and controls. Cross-sectional study? Give the eligibility criteria and the sources and methods of selection of participants
		(b) Cohort study? For matched studies, give matching criteria and the number of exposed and unexposed. Case-control study? For matched studies, give matching criteria and the number of controls per case
Variables	7	Clearly define all outcomes, exposures, predictors, potential confounders, and effect modifiers. Give the diagnostic criteria, if applicable
Data sources/measurement	8*	For each variable of interest, give the sources of data and details of methods of assessment (measurement). Describe the comparability of assessment methods if there is more than one group
Bias	9	Describe any efforts to address potential sources of bias
Study size	10	Explain how the study size was arrived at
Quantitative variables	11	Explain how quantitative variables were handled in the analyses. If applicable, describe which groupings were chosen and why
Statistical methods	12	(a) Describe all statistical methods, including those used to control for confounding
		(b) Describe any methods used to examine subgroups and interactions
		(c) Explain how missing data were addressed
		(d) Cohort study? If applicable, explain how the loss to follow-up was addressed. Case-control study? If applicable, explain how the matching of cases and controls was addressed. Cross-sectional study? If applicable, describe the analytical methods taking account of the sampling strategy
		(e) Describe any sensitivity analyses
Results		
Participants	13*	(a) Report the numbers of individuals at each stage of the study, e.g., numbers potentially eligible, examined for eligibility, confirmed eligible, included in the study, completing follow-up, and analyzed
		(b) Give the reasons for non-participation at each stage
		(c) Consider the use of a flow diagram
Descriptive data	14*	(a) Give the characteristics of study participants (e.g., demographic, clinical, social) and information on exposures and potential confounders
		(b) Indicate the number of participants with missing data for each variable of interest
		(c) Cohort study? Summarize the follow-up time (e.g., average and total amount)
Outcome data	15*	Cohort study? Report the numbers of outcome events or summary measures over time
		Case-control study? Report the numbers in each exposure category or summary measures of exposure
		Cross-sectional study? Report the numbers of outcome events or summary measures
Main results	16	(a) Report the numbers of individuals at each stage of the study, e.g., numbers potentially eligible, examined for eligibility, confirmed eligible, included in the study, completing follow-up, and analyzed
		(b) Give the reasons for non-participation at each stage
		(c) Consider the use of a flow diagram
Other analyses	17	Report the other analyses done, e.g., analyses of subgroups and interactions and sensitivity analyses
Discussion		
Key results	18	Summarize the key results with reference to the study objectives
Limitations	19	Discuss the limitations of the study, taking into account the sources of potential bias or imprecision. Discuss both the direction and the magnitude of any potential bias
Interpretation	20	Give a cautious overall interpretation of results considering the objectives, limitations, multiplicity of analyses, results from similar studies, and other relevant evidence
Generalizability	21	Discuss the generalizability (external validity) of the study results
Other information		
Funding	22	Give the source of funding and the role of the funders for the present study and, if applicable, for the original study on which the present article is based

Appendix B

**Table 4:** Questionnaire

Please express your agreement or disagreement regarding the following statements:	Agree	Disagree	Neutral
1. Childbirth is a process that should not be interfered with, unless medically necessary			
2. Skin-to-skin, mother-child contact is easier to perform when birth is natural or vaginal birth			
3. After a vaginal birth, the mother's recovery is easier and faster			
4. Vaginal birth increases a woman's self-confidence			
5. The mother-child emotional relationship after vaginal birth is better			
6. Fear of pain during childbirth influences the way of birth			
7. Children born vaginally have a better adaptation to extrauterine life			
8. Scheduled cesarean section is associated with the risk of prematurity of the newborn			
9. The drugs used for anesthesia in cesarean surgery are harmful to the fetus			
10. It is the pregnant woman's right to choose the method of birth, but her choice must be an informed and medically approved one			
11. Cesarean section is a life-saving intervention for women and children when there are complications during pregnancy and at birth			
12. Pain during childbirth is one of the indications for cesarean section			
13. Cesarean section on request is more accessible in a private environment			
14. Vaginal birth causes a decrease in sexual satisfaction			
15. Children born by cesarean section are healthier than those born vaginally			
16. The child's non-adaptation to extrauterine life is more frequent in the case of cesarean section			
17. Newborn babies born by cesarean section are more intelligent			
18. Vaginal birth is preferable because the scars after cesarean section are unsightly			
